# The Pathogenesis of Papilledema: Review of the Literature and a New Hypothesis

**DOI:** 10.1007/s11538-025-01465-7

**Published:** 2025-06-25

**Authors:** David N. Levine, Ari I. Rapalino

**Affiliations:** 1 Department of Neurology, New York University Grossman School of Medicine, 415 East 37 th Street, # 26L, New York, NY 10016 USA; 2 Department of Hematology/Oncology, Boston Children’s Hospital, 300 Longwood Ave., Boston, MA 02115 USA

**Keywords:** Papilledema, Acute glaucoma, Biomechanics, Pathogenesis, Axonal transport

## Abstract

Since the first description of swelling of the optic nerve head in patients with increased intracranial pressure, our understanding of its pathogenesis has undergone significant changes. Early theories postulated that the swelling was caused by excessive extracellular fluid, but these views were disproved when electron microscopy showed that the swelling arose from dilated optic nerve axons, and autoradiography demonstrated blocked axonal transport at the posterior lamina cribrosa. This led to the currently prevailing view that the axonal swelling is caused by the damming back of axoplasm. However, this theory cannot account for the extent of swelling, its rate of development, and the variety of morphological changes in papilledema. It also cannot explain the differing patterns of swelling in papilledema and acute glaucoma despite identically located blockages of axonal transport. We conducted a biomechanical analysis, in which we calculated the stresses induced in a cylindrical nerve by external compression and the effect of these stresses on the nerve’s axons and the axoplasm within them. We propose a new theory in which the axial gradient of tissue pressure causes displacement of axoplasm from the extraocular to the intraocular segment of the nerve, accounting for the intraocular axonal swelling. In addition, a sharply localized axial shear stress disrupts the axonal cytoskeleton to block axonal transport. Although the pressure gradient and the shear stress are both caused by the external compression of the nerve, they differ in their relative magnitudes across the nerve cross-section. The proposed hypothesis resolves the difficulties with the damming back hypothesis.

## Optic Nerve Anatomy

**Fig. 1:**
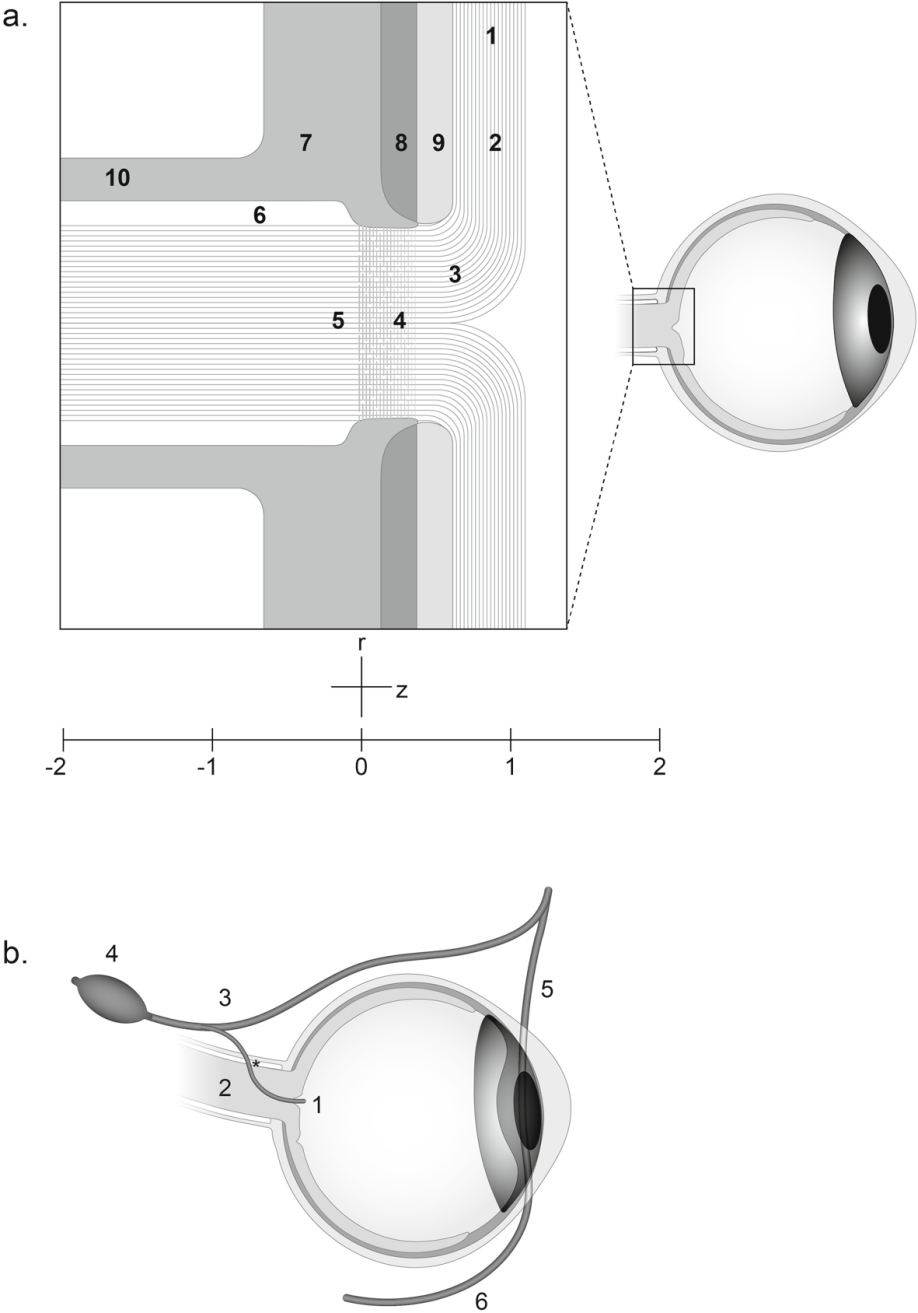
a. Schematic representation of the anatomy of the optic nerve and its adjacent structures. Numbers 1–5 represent successive segments of individual ganglion cell axons: 1. retinal nerve fiber layer; 2. peripapillary area; 3. prelaminar optic nerve; 4. laminar optic nerve traversing the lamina cribrosa; 5. retrolaminar optic nerve. Segments 1–4 are intraocular and segment 5 is extraocular except for it most anterior extremity. Numbers 6–10 are adjacent structures: 6. subarachnoid space filled with cerebrospinal fluid; 7. sclera; 8. choroid; 9. retina; 10. dura. **b**. Schematic representation of the course of the central retinal vein carrying blood from the retina: 1. intraocular central retinal vein; 2. intraneural extraocular central retinal vein. Asterisk denotes central retinal vein crossing the subarachnoid space to become extraneural. 3. superior ophthalmic vein; 4. cavernous sinus; 5. angular branch of the facial vein; 6. facial vein

Papilledema, to be defined below, distorts the anatomy of the optic nerve. A brief review of the relevant normal anatomy of the nerve may prove helpful in understanding many of the terms used in this paper. This section should be read with reference to Fig. 1.

The optic nerve is a cylindrical structure that contains approximately one million axons. The axons are cylindrical tubes with a mean diameter of 0.72µ but with a skewed distribution such that diameters range from 0.25 to 2.5 µ (Mikelberg et al. 1989). The wall of each axon, a bilipid membrane called the axolemma, is extremely thin, measuring about 8 nm in thickness. The tubes are filled with axoplasm, a gel-like substance whose rheology is detailed below. Each axon originates from the cell body of a ganglion cell of the retina. The axons travel in the retinal nerve fiber layer (Fig. 1a), which constitutes the innermost layer of the spherical shell that is the wall of the eye. Internal to this layer is the vitreous fluid, which fills the posterior portion of the cavity of the shell. In the retinal nerve fiber layer, the axons converge towards the optic disc, also known as the head of the optic nerve or the optic papilla (Fig. 1a). The optic disc is a circular area approximately 1.8 mm in diameter at the back of the eye that forms a cylindrical hole in the wall of the eye, about 1 mm deep, allowing the axons to exit the eye. The axons enter the optic disc to form the intraocular portion of the optic nerve. The intraocular nerve has three parts—the prelaminar, the laminar, and the most anterior part of the retrolaminar segment. As the axons from the retinal nerve fiber layer enter the optic disc, they traverse the prelaminar segment which consists of the axons and their surrounding glial cells with little to no lateral constraint. They then traverse the laminar portion of the intraocular optic nerve, which is bordered circumferentially by the sclera, a relatively rigid collagenous structure that provides significant lateral constraint. The laminar portion of the intraocular nerve is so named because of the lamina cribrosa—a series of planar meshes orthogonal to the course of the axons. Each mesh consists of collagenous fibers that originate from the scleral border. A bundle of axons traverses each pore of a mesh, and the pores of successive meshes are aligned so that the axons maintain their axial course. Upon crossing the posterior border of the lamina cribrosa, the axons, now referred to as retrolaminar, acquire a myelin sheath and exit the eye to lie in the orbital, or extraocular portion of the optic nerve. Here the axons are surrounded by the meningeal layers of the brain: the pia, arachnoid and dura. Notably, there is a space between the arachnoid and the pia, filled with cerebrospinal fluid, that surrounds the entire retrolaminar optic nerve and communicates with the fluid surrounding the brain and spinal cord. The orbital optic nerve extends posteriorly for about 25 mm, at which point the axons enter and traverse the optic canal, a bony passageway approximately 9 mm long. They then occupy the intracranial space for a distance of 16 mm before forming in succession the 9 mm long optic chiasm and the 5 mm long optic tract at the base of the brain. After this approximately 65 mm extraocular course, the axons enter and synapse in the lateral geniculate nucleus of the thalamus.

The anatomy of venous blood flow from the retina is also relevant, as it has figured prominently in theories of the pathogenesis of papilledema. The central retinal vein (Fig. 1b) receives blood from the many small venules of the retina and leaves the eye through the optic disc, where it is centrally located. After leaving the eye, it continues posteriorly to lie within the extraocular nerve for a distance of 10 mm after which it exits the nerve, crossing the subarachnoid space—where it is subject to the pressure of the cerebrospinal fluid—and the dura to lie freely in the orbit where it empties into the superior ophthalmic vein. The latter, in turn, courses both posteriorly and anteriorly. Posteriorly, it empties into the cavernous sinus, a large venous structure at the base of the brain. Anteriorly, it communicates with the angular branch of the facial vein. Although blood flow is typically in the posterior direction, the blood can flow anteriorly should an obstruction of the cavernous sinus arise**.**

## Historical Introduction and Review of the Literature

In 1860 Albrecht von Graefe described a patient with a brain tumor that had caused paralysis, recurrent seizures, and blindness with dilated pupils. Ophthalmoscopic examination showed swelling of the head of the optic nerve. At autopsy the optic nerves were normal except for the most anterior portions, which projected forward into the eye, and showed “serous infiltration as well as hypertrophy of the cellular elements.” Later, having seen a similar picture in three other patients with brain tumor, von Graefe (1866) postulated that the tumors compressed the cavernous sinus, causing stasis in the central retinal vein that was exacerbated by the constraint imposed on it by the unyielding scleral ring surrounding the laminar part of the intraocular optic nerve. The venous stasis led to the serous infiltration and swelling in the head of the optic nerve, which von Graefe called “Stauungspapille”, later translated as “choked disc” (Allbutt 1872). Von Graefe’s theory of pathogenesis was later rendered implausible due to the discovery that the superior ophthalmic vein, into which the central retinal vein emptied, anastomosed freely with the facial vein (Fig. 1b), thereby affording a pathway for venous return from the eye that bypassed the cavernous sinus, so that compression of the latter would be unlikely to cause stasis in the central retinal vein (Sesemann 1869). Schmidt (1869) proposed an alternative theory of pathogenesis. Building upon Schwalbe’s (1869) discovery that the subarachnoid space of the brain communicated with the subarachnoid space surrounding the optic nerve, Schmidt injected blue dye into the cerebral subarachnoid space of an exsanguinated dog and followed the dye into the subarachnoid space surrounding the optic nerve and from there into the nerve itself in the area of the lamina cribrosa (LC). He proposed that elevated intracranial pressure (ICP) forced the flow of cerebrospinal fluid (CSF) into the nerve causing swelling of the optic disc (Fig. 1a). While the majority of early investigators agreed that swelling of the head of the optic nerve was caused by mechanical compression in some form, either of the venous drainage of the eye at some point in its course, or of the optic nerve itself from the CSF that surrounded it and infiltrated its interstitial spaces, there were notable dissenters. In particular, Gowers (1879) asserted that any swelling of the head of the optic nerve was due to inflammation. As a result, for over 30 years, well into the first decade of the twentieth century, disc swelling associated with brain tumor was called “optic neuritis” in the English language literature. Quite early, however, Hughlings Jackson (1871) cautioned that ophthalmoscopic “optic neuritis” was not a single disease; some cases had early loss of vision, while in others vision was normal. In 1908 Parsons (1908) coined the term “papilledema” to refer to the marked disc swelling—more than 2 diopters—associated with increased ICP, distinguishing this severe swelling from the “papillitis of moderate degree due to a great variety of causes.”

Paton and Holmes (1911) brought considerable clarity to both pathogenesis and terminology with their study of the pathology of disc swelling associated with brain tumors. They noted and discussed four groups of theories: (1) inflammation of the head of the optic nerve; (2) spreading of brain edema surrounding a tumor to the optic nerve; (3) an abnormal vasomotor reaction of the retinal blood vessels caused by a brain tumor; (4) a mechanical effect of increased ICP. They demonstrated conclusively that there was little or no inflammation associated with the disc swelling. They presented cogent arguments against the second and third sets of theories and concluded that the mechanical effects of increased ICP were of primary importance. They used Parsons’ term “papilledema” to refer to any swelling of the disc caused by increased ICP, while “optic neuritis” referred to swelling caused by inflammation. It was soon realized (Paton 1936) that the two conditions could be distinguished clinically. Papilledema was painless and associated with normal vision until late in its course, whereas optic neuritis was often painful with early loss of vision. Paton and Holmes proposed their own mechanical theory of pathogenesis. They believed that increased ICP in the subarachnoid space surrounding the retrolaminar optic nerve was transmitted to the central retinal vein as it crossed this space (Fig. 1b), leading to increased intravascular pressure in the intraneural central retinal vein. The elevated ICP also increased tissue pressure in the retrolaminar optic nerve surrounding the vein, so that the transmural pressure remained normal. In contrast, the tissue pressure surrounding the prelaminar central retinal vein was determined by the intraocular pressure (IOP), which was normal. In this manner the transmural pressure of the prelaminar central retinal vein was elevated, leading to transudation of fluid (“lymph”). Under ordinary circumstances such fluid would drain into the subarachnoid space along the perivascular spaces, but this drainage was obstructed by the elevated pressure in the subarachnoid space (“lymph stasis”). Hence extracellular fluid accumulated around the axons of the head of the optic nerve.

The theory of Paton and Holmes took hold and was not seriously challenged for more than 60 years. The creation of animal models of papilledema in primates with anatomy closely resembling that of humans (Glew et al. 1958; Hayreh 1964) enabled further understanding of pathogenesis by means of new technology such as electron microscopy and new techniques such as autoradiography. The advent of the electron microscope allowed re-examination of the pathology of papilledema at higher resolution. Tso and Hayreh (1977a) induced increased ICP and papilledema in rhesus monkeys by inflating an intracranial balloon. They found that papilledema was predominantly swelling of axons in the prelaminar optic nerve with only a very modest contribution from extracellular fluid. Earlier, Schutta and Hedges (1971) had essentially similar results but attempted to reconcile their findings with the theory of Paton and Holmes by attributing the swelling of the axons to fixation artifact, whereby, as a result of incomplete perfusion by fixative, the still surviving axons could imbibe the extracellular fluid. Tso and Hayreh (1977b), however, showed that horseradish peroxidase, a small molecule used as a marker of extracellular space of the central nervous system, did not enter the swollen prelaminar axons when injected intravenously shortly before perfusion with fixative.

At the same time that electron microscopy demonstrated that papilledema involved swelling of axons rather than expansion of the extracellular space by fluid extravasated from the microvasculature, studies of axoplasmic transport suggested a possible mechanism of the axonal swelling. Tso and Hayreh (1977b) injected tritiated leucine into the eyes of rhesus monkeys, which was taken up by the retinal ganglion cells and incorporated into protein. By means of autoradiography, they traced the course of the radiolabeled protein down the axon in both normal monkeys and in monkeys that had developed papilledema after inflation of an intracranial balloon. In normal monkeys the radioactivity appeared in the retinal nerve fibers within a day and proceeded anterograde (away from the ganglion cells of origin in the retina), so that nearly all of the radioactivity passed through the LC after four days. In the monkeys with papilledema radioactivity accumulated at the LC, reaching a peak at three days, and was still abnormally concentrated there twelve days after injection. Tso and Hayreh concluded that in papilledema there is a localized block of both fast and slow axoplasmic transport at the LC. Hayreh (1977, 2016) further stated that increased CSF pressure in the subarachnoid space led to increased tissue pressure in the optic nerve, which impeded the flow of axoplasm away from the cell body. The location of the block at the LC was admittedly a mystery, but he speculated that it was the “meeting point” between IOP and ICP. Ordinarily, the former is higher than the latter, facilitating axoplasmic flow away from the eye, but in the presence of elevated ICP the opposite may be true, leading to damming back of the axoplasm and swelling of the axons proximal to the block. Later, the swollen axons could compress capillaries and veins on the surface of the disc and in the prelaminar region, leading to transudation of fluid, which contributed secondarily to the swelling.

The theory that papilledema is primarily an axonal swelling caused by impaired axoplasmic flow has largely supplanted the theory that increased intracranial pressure acts primarily on the prelaminar central retinal vein and other vessels of the optic disc to produce an excess of extracellular fluid. With the discovery of several mechanisms transporting a variety of axoplasmic components at different rates (Maday et al. 2014), the term “axonal transport” has largely replaced the terms “axoplasmic flow” and “axoplasmic transport”. These three terms will be employed synonymously in this paper.

## Critique of the Currently Prevalent Theory

There are several problems with the prevailing theory that axonal swelling in papilledema is caused by the damming back of axoplasm due to blocked axonal transport:

### Identically Located Blocks of Axonal Transport Do Not Result in Identical Patterns of Axonal Swelling

If increased intracranial pressure leads to papilledema by blocking axonal transport and thereby causing swelling of the prelaminar optic nerve axons, then one would expect similar axonal swelling whenever such a block of axoplasmic flow develops, even if it is not caused by increased intracranial pressure but rather by another pathophysiologic state. Several investigators have shown that acute elevation of IOP, like elevation of ICP, creates a localized block of both anterograde and retrograde (towards the ganglion cell of origin in the retina) axonal transport at the posterior border of the LC (Anderson and Hendrickson 1974; Minckler et al. 1977a, 1976; Quigley and Anderson 1976; Tso and Hayreh 1977b). Although prelaminar optic nerve axonal swelling occurs both in animal models where IOP is acutely raised and in human cases of acute glaucoma (Elschnig 1928), its evolution and duration differ markedly from the axonal swelling associated with increased ICP. The swelling of increased ICP begins after a delay of one or more days, increases progressively over time, and persists for many weeks or more. There is no swelling of the retrolaminar optic nerve axons; in fact, scattered collapse of some of these axons has been reported (Tso and Hayreh 1977a). In contrast, the prelaminar axonal swelling associated with acute elevation of IOP occurs within a day of elevating IOP, lasts for a few days, during which it is never pronounced, and then subsides, such that after a week the prelaminar axons are no longer swollen (Zimmerman et al. 1967). Rather, it is the retrolaminar optic nerve axons that undergo more severe and long-lasting swelling. Why should a block of axonal transport at the LC have such different manifestations in the two conditions?

It could be argued that elevated pressure suppresses damming back of fluid caused by a block of axonal transport. Thus, in acute glaucoma, the elevated IOP might suppress the prelaminar damming back that would otherwise occur with blockage of anterograde axonal transport, and, in papilledema, the elevated ICP might suppress the retrolaminar damming back that would otherwise result from blockage of retrograde axonal transport.

This argument fails for two reasons. First, it cannot explain the dynamics of prelaminar axonal swelling in acute glaucoma—how, with an unchanging level of elevated IOP, prelaminar axonal swelling develops in a day, persists for a few days, and then subsides and disappears. If the initial prelaminar axonal swelling results from blocked anterograde axoplasmic flow, despite the presence of elevated IOP, why does the swelling then subside, even though the block persists and the elevated IOP remains unchanged? Second, it has been shown that papilledema occurs not only with elevated ICP and normal IOP, but also with normal ICP and reduced IOP (ocular hypotonia) (Dellaporta 1955; Ectors and Begaux-VanBoven 1941). The papilledema of reduced IOP, like that of increased ICP, is associated with a block of both anterograde and retrograde axonal transport at the LC (Minckler et al. 1977b), and the pathology of the two conditions is identical in showing severe axonal swelling limited to the prelaminar region (Minckler and Tso 1976). But, in the papilledema of ocular hypotonia, there is no elevation of ICP that would suppress damming back and swelling of the retrolaminar axons from blocked retrograde axoplasmic flow. Yet, such swelling does not occur.

### The Volume of Axoplasm Delivered by Axonal Transport is Insufficient to Account for the Extent of the Axonal Swelling in Papilledema and the Rate at Which It Develops

Weiss and Hiscoe (1948), after constricting a peripheral nerve with an arterial sleeve for seven months, found that the excess volume that had accumulated in the region abutting the constricting sleeve was sufficient to fill less than a 2 mm length of an axon of normal diameter. In papilledema an axon can be swollen up to 20 times its normal diameter. The axial distance over which this swelling occurs is not known, but the course of a swollen axon is often sinuous, extending through the portions of the LC opposite the choroid and retina into the prelaminar and peripapillary region (Fig. 1a). To take a conservative estimate, if the 20-fold swelling is only 0.1 mm in axial length, it would contain enough axoplasm to fill a normal axon for a length of 40 mm.

Correspondingly, the velocity of axonal transport is too slow to account for the evolution and magnitude of the axonal swelling in papilledema. Weiss and Hiscoe removed a sleeve that had compressed a nerve and narrowed its axons. They studied the wavefront of axoplasm that flowed longitudinally from the region proximal to the constriction into the previously constricted region and estimated its velocity to be between 0.5 and 2 mm/day. As the flow of axoplasm was into constricted axons with diameters less than one third of normal, an equal volume flowing through axons of normal diameter would have a velocity ranging from 0.06 to 0.24 mm/day. Papilledema is generally detectable within a few days of elevated ICP (or marked depression of IOP) and reaches a maximum within a few weeks of its detection, at which time axons may be swollen to 10–20 times their normal diameter. Even if we use the fastest equivalent velocity of 0.24 mm/day, it would require more than 160 days of blocked axoplasmic flow to swell a 100μ length of axon to 20 times its normal diameter. (The number of days is equal to the volume that must be filled divided by the volume delivered per day. If the normal axon radius is $\hat{R}$, the volume to be filled is $\pi \left( {20\hat{R}} \right)^{2} \left( {.1mm} \right)$ and the volume delivered per day is $0.24\frac{mm}{{day}}\pi \hat{R}^{2}$. The quotient is $\frac{40}{{0.24}} = 167$ days).

### The Morphology of Axons Affected By a Block of Axonal Transport Does Not Account for the Variety of Morphological Changes of Axons in Papilledema

Weiss and Hiscoe (1948) found that when a nerve was compressed concentrically by an arterial sleeve, the axons became increasingly distorted as they approached the constriction. Nearest to the constriction there was periodic gross ballooning of the axons. The balloons could herniate through the axon membrane to form blind pouches (axonal spheroids). More proximally, further from the constricting sleeve, the ballooning eased off into gentler beading. Telescoping of the axons also occurred, a combination of beading with longitudinal compression of the axon, so that one bead invaginated the next most distal one, “resembling the configurations assumed by a rubber tube that is being stripped off a closely fitting rod.” Smaller fibers became tightly helically coiled “like accommodating a flexible rope in a cylindrical container that is too short.” The internodes became progressively shorter as the distance from the constricting sleeve decreased. Their interpretation was that the constricting arterial sleeve imposed a bottleneck on the anterograde flow of axoplasm, so that it accumulated just proximal to the constriction, “comparable to a traffic jam”. The physical effect on the axon was a longitudinal compression starting from the level of resistance at the constriction and working backward.

Reports on the pathology of papilledema, in both humans (Paton and Holmes 1911; Tso and Fine 1976) and primates (Minckler and Tso 1976; Tso and Hayreh 1977a), at both light and electron microscopic levels of resolution, have reported none of these distortions. However, these studies were not designed optimally to detect such distortions, which are best seen when one can follow the longitudinal course of a single axon. Nevertheless, organelles such as mitochondria, vesicles, and electron-dense bodies often accumulate in the ballooned pouches near a site of blocked axonal transport (Klinic et al. 2008; Yong et al. 2021), and these are easily seen in routine cross-sections. Indeed, the optic nerve head in papilledema shows occasional axons with cross-sections that are often irregular in outline, containing numerous mitochondria, that appear either normal, shrunken or swollen, as well as electron dense bodies (Minckler and Tso 1976; Tso and Fine 1976; Tso and Hayreh 1977a). The axons themselves may be shrunken or swollen to various degrees. More common, however, are grossly dilated axons with a smooth cylindrical border that contain a few scattered mitochondria and neurofibrils or a watery axoplasm with few or no cytoplasmic organelles. There appears to be no correlation between the degree of swelling and the presence of accumulated organelles.

## A Biomechanical Model of the Compressed Optic Nerve and Its Axons

Although it is generally believed that papilledema is the result of compression of the optic nerve by a surrounding sleeve of CSF at elevated pressure, the nature of this mechanical effect, as discussed above, is not clear. It therefore seems worthwhile to examine the effects of such a sleeve of increased pressure, which surrounds the nerve through its entire retrolaminar course, but ends abruptly at the posterior margin of the LC, where the subarachnoid space ends (Fig. 1a). If we can determine the nature and the distribution of the mechanical stresses created in the nerve by such a pressure sleeve, it may be possible to predict the effects of these stresses on the axons of the nerve and on the fluidity and structural integrity of the axoplasm that they contain. To date, no such biomechanical analysis has been done.

**Fig. 2:**
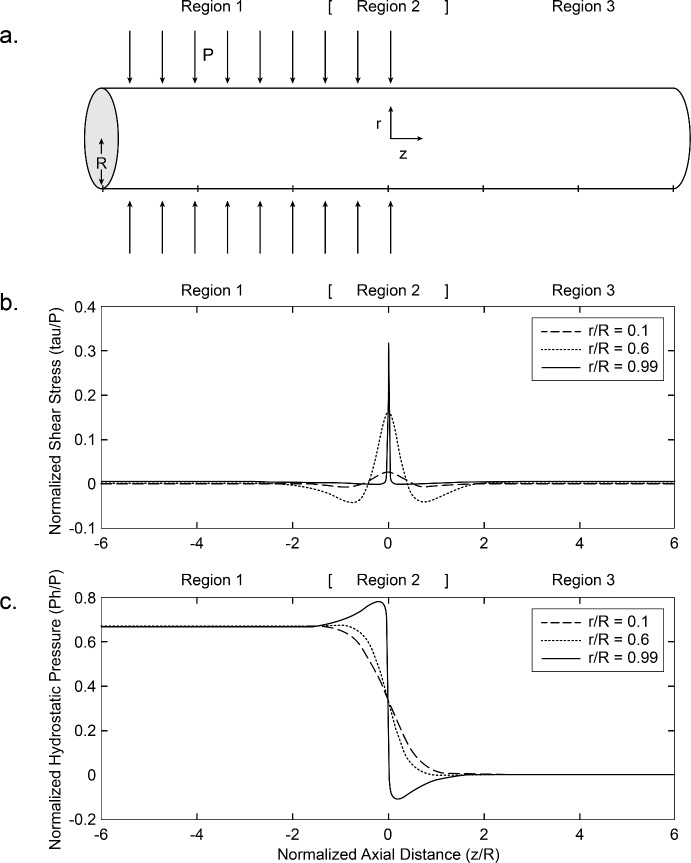
a. A model of the optic nerve as a long solid cylinder of radius ${\text{R}}$ with its central axis corresponding to the ${\text{z}}$-axis. The perpendicular distance of a point in the cylinder from the central ${\text{z}}$-axis corresponds to the ${\text{r}}$ coordinate. The origin, depicted by the junction of the small labelled ${\text{r}}$ and ${\text{z}}$ axes in the center of the cylinder, is at the center (${\text{r}} = 0)$ of the cross-section, at the transition point (${\text{z}} = 0)$ between the compressed (${\text{z}} < 0)$ and the uncompressed (${\text{z}} > 0)$ portions of the nerve. Anatomically, the transition point corresponds to the posterior border of the lamina cribrosa and the anterior border of the subarachnoid space. The compressed portion includes region 1 and the right half of region 2 and corresponds anatomically to the retrolaminar optic nerve axons. The uncompressed region includes the right half of region 2, which corresponds anatomically to the laminar, prelaminar and peripapillary segments of the optic nerve axons, and region 3, which corresponds to the retinal nerve fiber layer. The normalized ${\text{z}}$ coordinates (z/R) are aligned with those of the middle and bottom panels. **b.** The shear stress $\tau_{rz}$ plotted against the normalized axial distance z/R, for the three values of ${\text{r}}$ noted in the legend. **c.** The tissue hydrostatic pressure plotted against z/R for the three values of ${\text{r}}$ noted in the legend

We adopt an admittedly simplified model of the optic nerve as a solid cylindrical structure, which is compressed over much of its length by an abnormally elevated external pressure $P$, but at a particular point, hereafter called the transition point, is abruptly released from that pressure (Fig. 2a). The transition point corresponds anatomically to the anterior termination of the subarachnoid space and the posterior margin of the lamina cribrosa. It is given the axial co-ordinate $z = 0$. The radial co-ordinate $r$ ranges from zero on the axis of the nerve to $R$ at the surface of the nerve. Negative values of z correspond to the retrolaminar, largely extraocular nerve, while positive values of z correspond to the intraocular part of the nerve and the retinal nerve fiber layer. The major simplification is that the retinal nerve fiber layer is spread over the inner surface of the spherical shell that is the wall of the eye and is not cylindrical until it enters the optic disc to become the prelaminar portion of the optic nerve. We believe that such a simplification is justifiable in the case of papilledema, as will be discussed later. The advantage of this simplification is that it is relatively straightforward to calculate analytically the distribution of the normal and the shear stresses induced in such a cylinder by the axisymmetric external compression $P$. The axisymmetry greatly simplifies calculations. There are only four non-zero forms of stress. Three are normal stresses that exert forces in a direction perpendicular to the co-ordinate planes of the cylinder: $\sigma_{r} \left( {r,z} \right)$, the radial normal stress, acts in a radial direction on planes perpendicular to the radial coordinate; $\sigma_{\theta } \left( {r,z} \right)$, the hoop stress, acts in a circumferential direction on planes created by sections of the cylinder that contain its central axis; and $\sigma_{z} \left( {r,z} \right)$, the axial normal stress, acts in the axial, or longitudinal, direction on cross-sectional planes of the cylinder. The fourth stress is a shear stress, $\tau_{rz} \left( {r,z} \right)$, which exerts force in the axial direction on planes perpendicular to the radial coordinate and in the radial direction on cross-sectional planes perpendicular to the axial coordinate. There are two equations of equilibrium involving these four stresses:

1 $$\frac{\partial {\sigma }_{r}}{\partial r}+\frac{\partial {\tau }_{rz}}{\partial z}+\frac{{\sigma }_{r}-{\sigma }_{\theta }}{r}=0$$

2 $$\frac{\partial {\tau }_{rz}}{\partial r}+\frac{\partial {\sigma }_{z}}{\partial z}+\frac{{\tau }_{rz}}{r}=0$$

The first equation expresses the balance of forces in the radial direction, and the second equation expresses the balance of forces in the axial direction. The problem to be solved is to determine the stresses that satisfy these equations and also satisfy the boundary conditions at the surface of the cylinder, namely: 1. There is a compressive normal stress of magnitude $P$ for $z < 0$ but no normal stress for $z > 0$; and 2. There is no shear stress.

Before presenting the results of the calculations, we must clarify the meaning of the compressive pressure $P$ in our model. First, as noted above, papilledema can occur without compressive pressure on the extraocular optic nerve if the IOP is pathologically lowered. It is the *difference* between ICP and IOP that is significant, not the absolute values of each. The importance of this pressure difference for the pathogenesis of papilledema (Henderson 1912; Parker 1911; Schutta & Hedges 1971), as well as glaucoma (Morgan et al. 1998, 1995; Yang et al. 2014), has been repeatedly emphasized. Thus, the external pressure $P$ in our model must be thought of as the difference between ICP and IOP. Second, we ignore the fact that, in the absence of any pathological external compression, the retrolaminar optic nerve is subject to the normal ICP, and the prelaminar optic nerve is subject to the normal IOP. These two normal pressures may not be the same. While they are not very different in the supine individual, the IOP may be considerably greater than ICP in the erect posture. We assume that these pressure differences either do not stress the optic nerve, or that the stresses that they cause are “physiological” in that they do not damage nerve tissue. Thus, our compressive pressure $P$ represents a *pathological* pressure difference between intracranial and intraocular pressures.

After we determine the normal and shear stresses in the compressed optic nerve as functions of the radial and axial co-ordinates in the nerve, we assign to each axon the stresses corresponding to its position in the nerve. We model each axon as a thin-walled cylindrical tube filled with axoplasm. The effect of these stresses on the axoplasm will depend, as will be shown below, on the radius of the axon and the rheology of the axoplasm.

There is relatively little data on the rheology of axoplasm, and the data that we have is largely from invertebrates. Squid axoplasm appears to be a gel, in particular a Bingham body (Buckingham 1921). At rest and unstressed, it is a solid with the consistency of a “stiff gel” (Rubinson & Baker 1979). When it is subjected to a shear force, at first it deforms like a solid, but when the shear force reaches a threshold—the yield stress—the gel liquifies, and the axoplasm behaves like a viscous liquid. For squid axoplasm, studied by Rubinson and Baker (1979), the yield stress was approximately 9.5 Pa, or 0.07 mm Hg. Once liquified, its viscosity was 140 Poise. In the absence of further information, we model the mammalian axoplasm as a Bingham body with similar yield stress and viscosity.

## Results

### The Internal Stresses Within the Cylindrical Optic Nerve Caused by an External Compressive Force of Elevated ICP Relative to IOP

The solution of the equations of equilibrium subject to the boundary conditions is carried out in detail in the appendix. The results are shown in appendix Eqs. 21–24 and again, in normalized form, in Eqs. 27–30. For our purposes, the average value of the three normal stresses, called the hydrostatic stress, is important. Its effect on a structural element, say a small cube of optic nerve tissue, is to increase or decrease its volume without otherwise distorting it. Because a compressive hydrostatic stress reduces the volume of such a structural element, it is assigned, by convention, a negative value. However, in everyday language, such a compressive hydrostatic stress is felt by the element on which it acts as a positive pressure. Therefore, the negative of the hydrostatic stress is called the tissue hydrostatic pressure $P_{h}$ (appendix Eqs. 25 and 33). Its importance lies in the fact that its axial gradient $\frac{{\partial P_{h} }}{\partial z}$ (appendix Eqs. 26 and 34), is a driving force for displacement of axoplasm along the axon.

Each form of stress shows three distinct regions along the cylinder (Fig. 2). Region 1 comprises all of the compressed portion of the nerve, except for a small segment belonging to region 2. It includes the entire extraocular nerve up to one cylinder radius from the transition point. Region 2 is centered at the transition point and extends one cylinder radius to each side of it. It includes the immediately extraocular portion of the nerve, which is compressed by the elevated CSF pressure, and the intraocular laminar and prelaminar nerve segments, as well as the peripapillary area, which are not compressed. Region 3 includes all of the uncompressed nerve exclusive of the small portion belonging to region 2 and corresponds to the retinal nerve fiber layer containing the ganglion cell axons that will converge onto the optic disc to form the optic nerve.

**Fig. 3:**
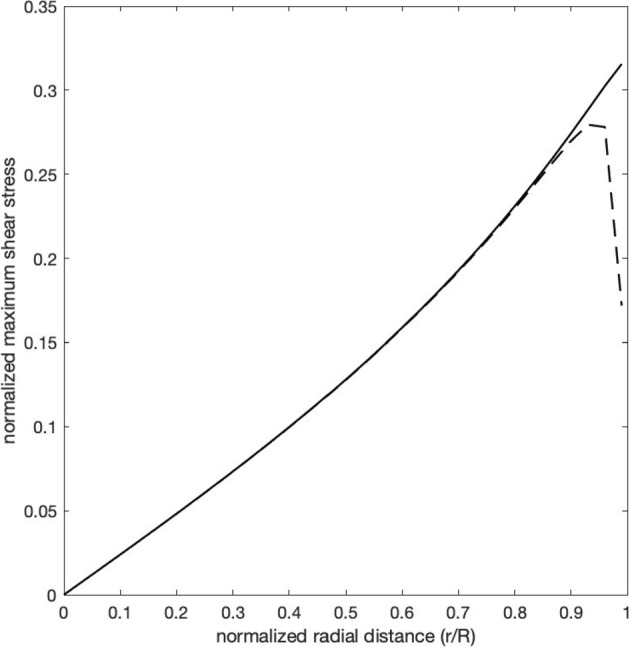
The magnitude of the maximal shear stress $\tau_{rz}$, located at $z = 0$, plotted as a function of $\frac{r}{R}$, the normalized radial distance from the central axis. The solid line is the plot for an abrupt transition from compressed to uncompressed nerve at the point $z = 0$. The dashed line is the plot for a transition zone of width $0.04{\text{R}}$ (36 $\mu$ for a nerve of radius 0.9 mm), over which the compression decreases linearly from ${\text{P}}$ to zero

We first consider the distribution of the shear stress. As shown in Fig. 2b, it is symmetric around the transition point, where it has its greatest magnitude and is positive, which means that it exerts a force towards the uncompressed part of the nerve (the positive $z$-direction). Away from the transition point it diminishes rapidly along the $z$ direction and, within one cylinder radius of the transition point, it becomes negative, which means that it exerts a force in the negative $z$ direction. The negativity gradually resolves and the stress disappears within two cylinder radii of the transition point. At the transition point, the magnitude of this shear stress increases nearly linearly with the radial distance from the center and is greatest just below the surface of the cylinder (Fig. 3—solid line).

**Fig. 4:**
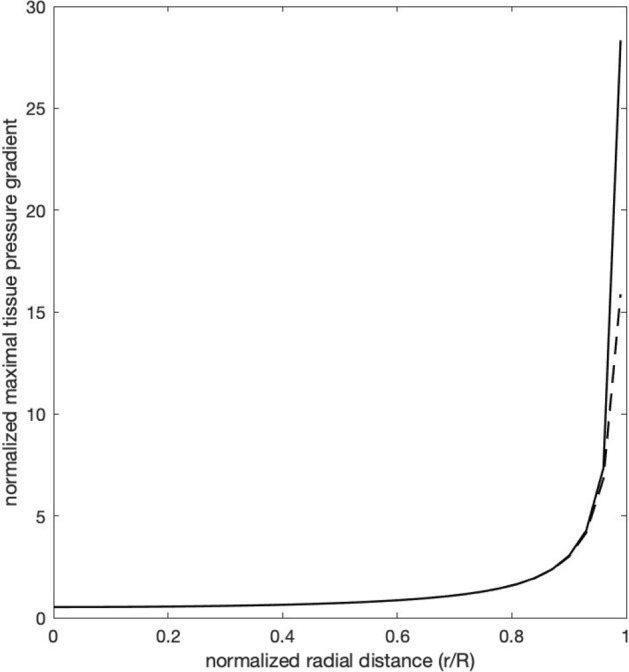
The maximal tissue hydrostatic pressure gradient, located at $z = 0$, plotted as a function of $\frac{r}{R}$, the normalized radial distance from the central axis. The solid line is the plot for an abrupt transition from compressed to uncompressed nerve at the point ${\text{z}} = 0$. The dashed line is the plot for a transition zone of width $0.04R$ (36 $\mu$ for a nerve of radius 0.9 mm), over which the compression decreases linearly from ${\text{P}}$ to zero

We next consider the distribution of the tissue hydrostatic pressure ${P}_{h}$ (Fig. 2c). In region 1 the radial and circumferential normal stresses are compressive with magnitude $P$, and the axial normal stress is zero. Therefore, $P_{h} = \frac{2P}{3}$. As one progresses towards the transition point, the changes in hydrostatic pressure are minimal, but, close to region 2, the hydrostatic pressure falls slowly and slightly in the central part of the nerve cross section, but rises slightly in the periphery. In region 2 the hydrostatic pressure falls with increasing rapidity as the transition point is approached. At the transition point $P_{h} = \frac{P}{3}$, and its rate of fall is maximal. The fall continues on the other side of the transition point, and reaches zero in region 3. The initial decline of the hydrostatic pressure occurs earlier—i.e., further from the transition point—and falls more gradually in the central part of the nerve cross-section than in the periphery, where, after the initial slight rise, the decline of hydrostatic pressure begins much closer to the transition point, and the decline is steeper than in the central part.^1^ The maximal hydrostatic pressure gradient, which occurs at the transition point, increases with the radial distance from the axis of the cylinder, at first rising slowly but then increasing dramatically in the peripheral 10% of the nerve (Fig. 4—solid line).

### Stresses on the Individual Axons of the Nerve and the Effect of These Stresses on the Axoplasm They Contain

An axon at radial coordinate $r$ coursing in the $z$-direction will be subject to the hydrostatic pressure ${P}_{h}$ and the shear stress ${\tau }_{rz}$ corresponding to the stresses calculated in the whole nerve for these particular coordinates. Thus, the stresses depicted in Fig. 2 can be thought of as the stresses experienced by individual axons along the $z$ -direction for each of the sampled radial co-ordinates. The existence of a negative gradient of tissue hydrostatic pressure in region 2 suggests the possibility that axoplasm within some axons may flow from the region of compression towards the uncompressed region of the nerve.

We now consider the condition for flow of a Bingham body through an axon modeled as a cylindrical tube. To avoid confusion with the radial co-ordinate $r$ of the optic nerve as a whole we use $\widehat{r}$ to denote the radial co-ordinate within an axon, which ranges from zero at the axis of the axon to $\widehat{R}$ at its surface.

**Fig. 5:**
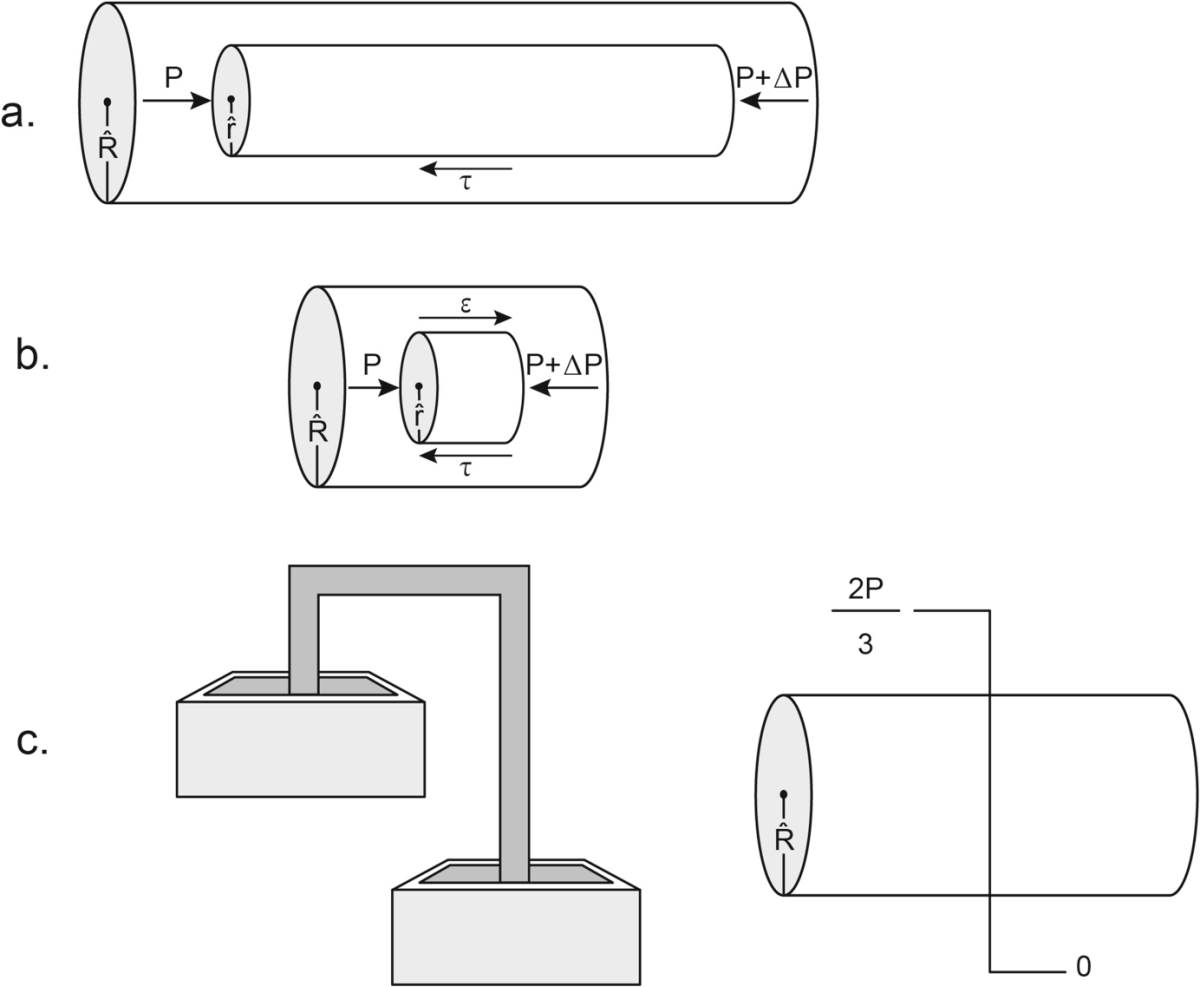
**a** Balance of forces on a cylindrical element of a Newtonian fluid of radius $\hat{r}$ flowing at constant velocity to the right through a cylinder of radius $\hat{R}$. The pressure difference $\Delta P < 0$ at the two ends, driving the fluid element to the right, is balanced by the leftward-directed viscous force $\tau$ applied to the surface by the surrounding fluid. **b** Balance of forces in an intraaxonal fluid element centered at the transition point from compressed to uncompressed nerve. Compared to part a there is an additional tangential force $\varepsilon$ to the right, which derives from the shear stress $\tau_{rz}$, induced by the external compression, that must be balanced by the viscous force $\tau$. **c** Comparison of a siphon with a compressed axon. In the siphon at the left, the upper and lower reservoirs differ in height and therefore in gravitational potential energy, but the fluid in each reservoir is subject to essentially the same atmospheric pressure. In the axon on the right there is a steep drop in tissue hydrostatic pressure at the transition point between compressed and uncompressed nerve, but, since the axon is horizontal, there is no change in gravitational potential energy

The condition for steady flow of a Newtonian fluid through a cylindrical tube can be derived from the balance of forces on a coaxial cylindrical element of fluid depicted in Fig. 5a. The difference of pressure $\Delta P_{h}$ is balanced by the shear stress $\tau$ exerted on the element by the surrounding fluid. Thus $\Delta P_{h} \left( {\pi \hat{r}^{2} } \right) + \tau \left( {2\pi \hat{r}\Delta z} \right) = 0$, and $\tau = \left( { - \Delta P_{h} /\Delta z} \right)\left( {\hat{r}/2} \right).$ The viscous shear stress thus varies linearly with the radial coordinate and is maximal at the wall of the tube, where it is given by $\tau_{wall} = \left( { - \Delta P_{h} /\Delta z} \right)\hat{R}/2$, where $\hat{R}$ is the radius of the tube. In a Bingham body flow will occur only if the shear stress at the wall of the axon exceeds the yield stress of the axoplasm. The condition for flow is thus:

3 $$\left( { - \Delta P_{h} /\Delta z} \right)\hat{R}/2 > f$$

where $f$ is the yield stress of the axoplasm. If this condition is met, flow will occur in the outer portion of the tube. However, because $\tau$ decreases from its value at the wall to zero at the center of the tube, there will be a radius $\hat{r}_{p}$, below which $\tau < f$, and the axoplasm within this radius will remain solid and be carried along with the peripheral flow as a solid plug. Since $\tau_{{r_{p} }} = f$ and $\frac{{\tau_{{r_{p} }} }}{{\tau_{wall} }} = \frac{{\hat{r}_{p} }}{{\hat{R}}}$, the fractional radial thickness of the plug is the ratio of the yield stress to the wall stress.

From Newton’s law of viscosity, $- dv/d\hat{r} = \frac{1}{\eta } \left( {\tau - f} \right)$, where $\eta$ is the dynamic viscosity and $dv/d\hat{r}$ is the radial velocity gradient. Substituting the previous expression for $\tau$, we have $dv/d\hat{r} = - 1/\eta \left[ {\left( {\left( { - \Delta P_{h} /\Delta z} \right)} \right)\frac{{\hat{r}}}{2} - f} \right]$. Integrating this expression, and using the no slip condition at the wall of the tube ($v\left( {\hat{R}} \right) = 0$) to calculate the constant of integration, we obtain an expression for the axial velocity as a function of the radial coordinate $\hat{r}$ when $\hat{r}_{p} < \hat{r} < \hat{R}$. The velocity of the central plug is obtained by setting the radial coordinate $\hat{r}$ equal to $\hat{r}_{p}$. Since $\hat{r}_{p} = \frac{{\tau_{{r_{p} }} }}{{ \tau_{wall} }}$ $\hat{R} = 2f/\left( { - \Delta P_{h} /\Delta z} \right)$, the velocity of the central plug of gel through the cylindrical axon, in a slightly rearranged form from that first given by (Buckingham 1921) is:

4a $$v = \frac{{\left( { - \Delta P_{h} /\Delta z} \right)}}{4\eta }\hat{R}^{2} \left( {1 - \frac{2f}{{ \hat{\user2{R}} { }\left( { - \Delta P_{h} /\Delta z} \right)}}} \right)^{2} \quad \text{if}\quad \hat{R} \left( { - \Delta P_{h} /\Delta z} \right) > 2f$$

4b $$\nu = 0\quad \text{if} \;\hat{R} \left( { - \Delta P_{h} /\Delta z} \right) < 2f$$

From the expressions above it is clear that the condition for flow to occur (Eq. 3) will most likely be met by those axons with a large radius $\widehat{R}$ and a steep gradient of tissue pressure $- \Delta P_{h} /\Delta z$.

Unlike conventional flow through a pipe, where the axial pressure falls linearly with distance along the pipe, in the axon the pressure gradient is sharply localized to the immediate neighborhood of the transition point. Outside this narrow neighborhood the hydrostatic pressure is constant, and the pressure gradient is zero. Thus, the condition for flow is determined at the transition point, where, instead of Eq. 3, the condition for flow is better expressed as

5 $$\left( { - \partial P_{h} /\partial z} \right)_{z = 0} \hat{R}/2 > f$$

As can be seen qualitatively from Fig. 2, and more quantitatively in Fig. 4, the maximal negative hydrostatic pressure gradient $\left( { - \partial P_{h} /\partial z} \right)_{z = 0}$ increases in magnitude the more peripherally the axon is located in the nerve. It is clear that flow of axoplasm across region 2, from the compressed to the uncompressed region of the nerve will be initiated at the transition point in large axons located near the surface of the nerve cross-section.

Further favoring the transition point as the site of initiation of flow is the behavior of the shear stress ${\tau }_{rz}$ induced in the optic nerve by the external compression (Fig. 2b). In deriving the condition of flow in Eqs. 1 and 5, the plug thickness ${\widehat{r}}_{p}$, and the plug velocity in Eqs. 4a and 4b, it was assumed that the only forces acting on a moving element of fluid were the pressure gradient pushing in the direction of lower pressure balanced by the viscous force exerted on the surface of the element by the surrounding fluid (Fig. 5A). This assumption does not strictly hold for the compressed nerve. At the transition point there is an additional positive shear stress, induced by the external compression (Fig. 2b). This positive shear stress is ultimately opposed and annulled by the negative shear stress seen in the side lobes of its plot against the $z$-coordinate, but that compensation has not yet fully occurred in the span over which the magnitude of the tissue hydrostatic pressure gradient is maximal. This situation is illustrated in Figs. 5b, in which we add to the balance of forces a small additional positive shear stress $\varepsilon$, which is the mean value of the stress ${\tau }_{rz}$ over the length of the fluid element. Thus, the viscous stress $\tau = \varepsilon + \left( {\left( { - \partial P_{h} /\partial z} \right)_{z = 0} \hat{r}/2} \right)$, and the condition for axoplasmic flow becomes $\left( { - \partial P_{h} /\partial z} \right)_{z = 0} \hat{R}/2 > f - \varepsilon$. The effect of $\tau_{rz}$ is equivalent to lowering the yield stress at the transition point from $f$ to $f - \varepsilon$. It thus facilitates the onset of axoplasmic flow, increases the initial velocity of the central plug, and reduces the thickness of the plug. These effects are maximal at the transition point toward the periphery of the nerve cross-section (Fig. 2b) and disappear once the mean value of ${\tau }_{rz}$ becomes zero at about one cylinder diameter from the transition point. The fact that the transition point is both the location of the maximum negative tissue pressure gradient and of the enhancing effect of the shear stress induced by the external compression emphasizes that liquefaction and displacement of the axoplasmic gel first occur at the transition point.

### The Nature of Axoplasmic Flow: The Axon as a Siphon

Once the condition for axoplasmic flow has been met, the behavior of the axon is analogous to that of siphon. In a siphon the flow is governed by the conservation of energy, expressed by Bernoulli’s principle:

6 $$P_{h} /\rho + gh + v^{2} /2 = C$$

In this equation $\rho$ is density, $h$ is elevation, $g$ is the gravitational acceleration, $v$ is the velocity of flow, and $C$ is a constant. The equation states that for a fluid flowing through a conduit the sum of its pressure energy, its gravitational potential energy, and its kinetic energy remains constant. In an ordinary siphon (Fig. 5c—left), a fluid gives up its gravitational potential energy for kinetic energy with no change in pressure energy. In our case (Fig. 5c—right), the axoplasm of a compressed axon gives up its pressure energy for kinetic energy with no change in gravitational potential energy.^2^

An ordinary siphon will continue to drain an elevated reservoir until it is either empty or the level of fluid in the outflow receptacle becomes equal to the level in the upper reservoir. Similarly, the axon will continue to drain axoplasm from its extraocular portion into its intraocular portion until either there is no more axoplasm left to drain or the pressure in the intraocular axon becomes equal to that in the extraocular axon.

Which of these two alternatives takes place—complete emptying of the extraocular portion of the axon, or partial emptying with equalization of pressure in the intraocular and extraocular axon segments—depends upon the surface tension behavior of the axon membrane. Once axoplasm traverses region 2 and enters region 3 it encounters axoplasm that is under no stress and therefore is in the form of a solid gel. This constitutes an impediment to further axial flow. In an ordinary siphon with conduits that have rigid walls such a clog causes flow to cease completely. The axon, however, has a relatively flexible membrane enclosing its axoplasm. Flow of axoplasm can occur in a radial as well as an axial direction. Unable to continue its axial flow, the axon will dilate, and the pressure within the axon may rise or fall depending on the surface tension behavior of the axon membrane. The relation between the radius $\hat{R}$ of the dilated axon and the pressure within it is governed by Laplace’s law, which, for a cylinder, takes the form

8 $$P = T\left( \hat{R} \right)/\hat{R}$$

where $T\left( \hat{R} \right)$ is the surface tension of the axon membrane. If the tension $T\left( \hat{R} \right)$ is constant, as in a soap bubble, or even if it rises more slowly than linearly with the radius of the axon, the pressure will decrease as the intraocular axon dilates. The axon will continue to dilate until emptying of the retrolaminar reservoir is complete. If $T\left( \hat{R} \right)$ rises with $r$ faster than linearly, the pressure in the axon will rise as the axon dilates, and if it reaches the value $2P/3$, which is its value in the compressed portion, flow will cease even if the extraocular part of the axon still retains axoplasm. In the prelaminar and peripapillary regions, the axons have minimal lateral support, and it is likely that $T\left( \hat{R} \right)$ rises sublinearly with the axon radius. By contrast, in the laminar region, there is stronger lateral support, offered by the sclera and the beams of the successive meshes of the lamina cribrosa. Hence, it is likely that expansion in the laminar segment will be limited. Overall, the situation resembles that of steady inflation of a balloon, which has an area of its wall that is weakened, with its elastic modulus lowered. The inflation will preferentially take place in the area with the weakened wall, which, in our case, is the prelaminar and peripapillary segments of the axon.

## A Hypothesis

The results above suggest an alternative hypothesis for the pathogenesis of papilledema. The axonal swelling that characterizes papilledema is the result of displacement of axoplasm from the compressed extraocular portion of the axon into its uncompressed intraocular portion. The mechanism of the displacement is as follows: Axoplasm in large axons under the transition point between the laminar and the retrolaminar optic nerve, particularly at the periphery of the nerve cross-section, flows into the uncompressed intraocular portion of the axon. The flow is driven by the steep negative gradient of hydrostatic pressure that exists at the transition point and is enhanced by the positive shear stress induced by the external compression. The axoplasm in the compressed extraocular axon is siphoned past the transition point into the intraocular axon. An everyday example of such siphoning of a Bingham body occurs when one squeezes an uncapped tube of toothpaste with his entire hand. Even though the pressure gradient is restricted to the edge of the hand closest to the spout, the toothpaste under the entire width of the hand flows out of the container. In the case of the axon, having entered the uncompressed intraocular region, the axial flow of axoplasm is blocked by the solid unsheared gel in the unstressed retinal nerve fiber layer, and the flow is diverted radially, dilating the axon. The flow continues until either the entire extraocular axon is emptied of its axoplasm or the pressure within the uncompressed axon rises to equal that of the compressed axon. Whether emptying will be complete or partial depends on the manner in which the surface tension of the axon membrane grows as the radius of the axon cylinder increases.

## Discussion

### The Localization of Papilledema

The hypothesis of axonal displacement is consistent with the localized nature of papilledema. Along the axial dimension, axonal swelling occurs primarily in the prelaminar and peripapillary regions of the intraocular optic nerve. The swelling does not extend into the retinal nerve fiber layer proximal to the peripapillary areas (Minckler and Tso 1976; Tso and Hayreh 1977a), because there is no pressure gradient sufficient to liquify the axoplasmic gel and sustain continued retrograde axial flow. The strong lateral support of the sclera and the beams of the lamina cribrosa limit the radial expansion of the posterior intraocular axons. Along the radial dimension, prelaminar axonal swelling is more prevalent and more severe in the periphery of the disc than in the center—a fact noted repeatedly in both clinical (Gowers 1879; Paton and Holmes 1911) and experimental (Minckler and Tso 1976) papilledema—because the axial pressure gradient needed to induce the flow of axoplasm has its highest magnitude peripherally. Along the circumferential dimension, the axons in the temporal sector of the optic nerve head have, on average, a smaller diameter than the axons of other sectors (Mikelberg et al. 1989). Because larger axons are more susceptible to axoplasmic displacement, this difference in average diameter may account for the relatively late and milder swelling in the temporal sector of the optic nerve head.

### Resolution of Critiques 3.1 and 3.2

#### The Papilledema of Increased ICP vs. the Papilledema of Increased IOP

As papilledema is not a direct consequence of a block of axonal transport, but rather the result of axoplasmic displacement, there is a reasonable answer to the question as to why the temporal pattern and severity of papilledema differ in these two conditions. Axoplasmic displacement occurs when there is an abnormal difference between ICP and IOP. When ICP is abnormally high relative to IOP, the axoplasm is displaced in a retrograde direction towards the ganglion cell body. The result is swelling of the prelaminar axons, which will progress and persist. In contrast, when IOP is abnormally high relative to ICP, axoplasm is driven across the lamina cribrosa in the opposite, anterograde direction, and the major and lasting swelling occurs in the retrolaminar region. This retrolaminar swelling is generally not detectable on ophthalmoscopic examination, but it has been observed both experimentally, as noted above, and in human pathology specimens (Knox et al. 2007). We propose that the early and transient prelaminar axonal swelling in acute glaucoma occurs because elevated IOP partially empties axoplasm from the susceptible retinal nerve fibers of the entire retina by inducing its anterograde displacement. The flow from these axons converges towards the optic disc. There the capacity to accommodate the large volume of anterograde flow is initially exceeded because of the lateral constraint imposed by the rigid sclera bordering the laminar segment of the nerve and possibly by a physiological partial constriction of axons at the LC (Hollander et al. 1995). The balance between anterograde axial and outward radial axoplasmic flow is shifted towards the latter, and the prelaminar axons swell. However, once the displaced axoplasm makes its way through the lamina cribrosa, the axonal swelling subsides.

#### The Extent of Swelling of the Optic Nerve Head and Its Rate of Development in Papilledema

The hypothesis of axoplasmic displacement can account for the massive axon swelling in papilledema. We note that the entire extraocular optic nerve, optic chiasm and optic tract is approximately 65 mm. long, and all of the axoplasm in that length of nerve is available for retrograde displacement to swell the prelaminar axons of the optic nerve. That axoplasmic displacement from the retrolaminar into the prelaminar optic nerve is the source of axonal swelling in papilledema receives further support from the observation that the retrolaminar optic nerve (region 1) not only does not show axonal swelling but also shows collapse of the myelin sheaths of some of the nerve fibers (Tso and Hayreh 1977a). We propose that the collapsed myelin sheaths are those that enveloped axons whose axoplasm has been displaced.

The axonal displacement hypothesis also predicts a time course for the development of papilledema that is reasonable in light of clinical and experimental evidence. Equation (5) gives the conditions under which axoplasm will flow toward the cell body. In an axon of radius $\widehat{R}$, axoplasm will flow only if $\left( { - \partial P_{h} /\partial z} \right)_{z = 0} \hat{R}/2 > f$. If the axon is sufficiently peripheral, that will always be possible, since the maximal pressure gradient at the transition point grows without bound as the outer border of the nerve cross-section is approached (Fig. 4 -solid line). Suppose the axon is so situated that$\left( { - \partial P_{h} /\partial z} \right)_{z = 0} \hat{R}/2 = 1.5f$. Then from Eq. (4a) the plug of axoplasm will flow with velocity$v=f\widehat{R}/12\eta$. If a prelaminar axon is swollen over a length $l$ to a width $k$ times its normal diameter, it will require displacement of the amount of axoplasm that originally occupied a length ${k}^{2}l$ of the normal retrolaminar axon. The time required will be $t=\frac{{k}^{2}l}{v}=\frac{12{k}^{2}\eta }{f{r}_{a}}l$. Using the values $f=9.5$ Pa and $\eta =14$ Pa-sec given by Rubinson and Baker (1979), and using $\widehat{R}=0.75$ microns as the radius of the axon, we find, taking the length of swelling to be 150 microns, that $v = .042$ microns/sec and $t = 3543k^{2}$ secs. If $k =$ 3, as might be the case in early papilledema, $t = 8.9$ hours. If $k = 20$, as might be the case when papilledema is maximal, $t=16$ days. These values are within the range of times observed clinically.

### Axial Shear Stress Causes the Localized Block of Axonal Transport in Papilledema

Blocked axonal transport at the posterior border of the LC has been solidly established in papilledema (Minckler et al. 1976; Tso and Hayreh 1977b). We have seen that compression of the retrolaminar optic nerve causes a localized gradient of the tissue pressure, which results in retrograde axoplasmic displacement, but the effect of this gradient on axonal transport is not obvious. However, when we consider that external compression also produces a shear stress that acts on longitudinally running structures, we may gain some clarity as to how axonal transport is disrupted. It is remarkable, and almost certainly not coincidental, that the localized defect in axonal transport coincides with region 2, the very same region to which this shear stress is confined. Axoplasm is a highly anisotropic substance with numerous longitudinally oriented structures—neurofilaments and neurotubules. The neurofilaments, a major component of the cytoskeletal structure of the axon, have radially oriented links to neighboring filaments (Yuan et al. 2012). A shear stress operating as in region 2 is optimally situated to cause longitudinal slippage of the filaments with respect to one another and to interrupt the radial linkages. The neurotubules play a major role in axonal transport; in particular, they are necessary for transport of neurofilament precursors that are integrated into the larger stationary matrix of neurofilaments along the axon (Yuan et al. 2012). The neurotubules are composed of parallel protofilaments that are laterally apposed to form the wall of a cylindrical tube. The seam between the protofilaments is a weak point that may splay open during the dynamic disassembly of the neurotubules (Mandelkow and Mandelkow 1994; Sui and Downing 2010). The orientation of the shear stress in region 2 may shear a neurotubular protofilament with respect to its immediate neighbors and disrupt the structure of the neurotubule. The shear in region 2 is thus oriented optimally to disrupt directly the axonal cytoskeletal framework and the tubules essential for axonal transport.

In addition to the direct effect of shear stress on these cytoskeletal structures, the shear stress may affect them indirectly by causing an influx of calcium ions, either by mechanically creating pores in the axolemma (Pettus et al. 1994; Pettus and Povlishock 1996) or, more indirectly, by activating sodium channels leading to depolarization that opens voltage-sensitive calcium channels (Wolf et al. 2001). The excess intracellular calcium may in turn activate proteases that break down the cytoskeleton, causing neurofilament compaction and loss of neurotubules. Among the activated proteases is calpain, which disrupts the immediate subaxolemmal actin-spectrin scaffolding (Büki and Povlishock 2006), a structure which is critical in preventing axonal beading (Ochs et al. 1997). While the mechanism by which beading occurs is still not known, it is possible that the intact actin-spectrin scaffolding sustains the balance of forces needed to maintain the cylindrical shape of the axon. On the one hand, the surface tension of the membrane inclines it to bead up in order to minimize the surface area. On the other hand, the actin-spectrin scaffolding provides tension that opposes the increase in curvature required for beading (Bar-Ziv and Moses 1994). With disruption of this scaffolding the surface tension forces are unopposed, and beading or ballooning of the axon may occur. This disruption may play an important role in leading ultimately to axonal degeneration. Finally, spectrin proteolysis affects the integrity of the mitochondrion by dissipating its membrane potential and opening the mitochondrial-membrane-permeability-transition pore. This leads to uptake of water and mitochondrial swelling (Büki and Povlishock 2006).

### Modification of the Model to Remove Singularities

Our model posits that the external force compressing the nerve transitions abruptly, i.e., discontinuously, from the elevated CSF pressure surrounding the retrolaminar optic nerve to the normal pressure surrounding the nerve in the lamina cribrosa. This creates two problems, which we deal with here.

First, the maximum shear stress increases linearly from the center of the nerve cross–section to the periphery, as shown in the solid line of Fig. 3. However, the compressive force of the CSF is perpendicular to the external boundary, so that the shear stress on the surface of the cylinder is zero. Thus, at the transition point, there is a discontinuity in the shear stress at the surface of the nerve.

Second, at the transition point, the model allows for a normalized axial gradient of tissue pressure, $\frac{R}{P}\left( { - \partial P_{h} /\partial z} \right)_{z = 0}$, that is infinite at the surface of the nerve, as shown in the solid line of Fig. 4. This implies that for any fixed increase in the external compression $P$, no matter how small (i.e., an infinitesimal increase in ICP, or an infinitesimal decrease in IOP), the non-normalized hydrostatic tissue pressure gradient $\left( { - \partial P_{h} /\partial z} \right)_{z = 0} = \frac{P}{R}\left[ {\frac{R}{P}\left( { - \partial P_{h} /\partial z} \right)_{z = 0} } \right]$ will increase without bound as the outer surface of the nerve is approached, since the expression in brackets becomes infinite. Thus, the condition for axoplasmic displacement $\hat{R}\left( { - \partial P_{h} /\partial z} \right)_{z = 0} > 2f$ will invariably be met in some axons, as long as they are close enough to the outer surface of the nerve cylinder, and papilledema will result. Clearly this is not the case. An infinitesimal rise in intracranial pressure will not result in papilledema, and the maximal normalized tissue pressure gradient, occurring just under the surface of the nerve cylinder, must be finite.

These deficiencies disappear if we replace the transition *point* with a short transition *zone*, over which the compressive external pressure falls linearly from $P$ to zero. This removes the discontinuity in the shear stress across the nerve cross section at the transition point (appendix Eq. 35). The dashed line of Fig. 3 illustrates this for a zone extending only $0.04R$, or 18 microns to either side of the former transition point, for a nerve that is 1.8 mm in diameter. The normalized shear stress at the transition point now rises almost linearly from the center of the nerve to reach a maximum at about 93% of the distance to the surface, at which point it begins to fall, at first slowly and then very rapidly to zero at the nerve surface.

The existence of a transition zone, no matter how limited its size, also removes the singularity in the axial gradient of tissue pressure. At the transition point, where the gradient is maximal, it now remains finite, even at the surface of the nerve. The dashed line of Fig. 4 illustrates this for a zone extending only $0.04R$, or 18 microns to either side of the former transition point, for a nerve that is 1.8 mm in diameter. The question then arises as to whether, given the small radii of the optic nerve axons, the product of the axon radius and the tissue pressure gradient will ever exceed twice the yield stress of the axoplasmic gel, so that retrograde flow of axoplasm will occur. If, in Fig. 4, the normalized tissue pressure gradient $\frac{R}{P}\left( { - \partial P_{h} /\partial z} \right)_{z = 0}$ has a finite maximum $M$, then the non-normalized gradient has as a maximum $\left( { - \partial P_{h} /\partial z} \right)_{z = 0} = \frac{P}{R}M$. In this expression $R$, the nerve radius, and M are fixed, but $P$ is not. If, at a given external pressure $P$ the maximum gradient $\left( { - \partial P_{h} /\partial z} \right)_{z = 0}$ is insufficient to induce flow of axoplasm in any axons, $P$ need only rise and thereby elevate the maximum gradient to the point where flow will occur. The degree to which $P$ must rise for axoplasmic displacement to occur in an axon of radius $\hat{R}$ under the maximum hydrostatic tissue pressure gradient is given by $\hat{R}\left( { - \Delta P_{h} /\Delta z} \right) = P\frac{{\hat{R}}}{R}M > 2f$; i.e., $P > 2\frac{R}{{\hat{R}}}\frac{f}{M}$. For example, if $M = 17$, as in the broken curve of Fig. 4, for a large peripheral axon of radius $\widehat{{R}}=1\mu$ in a nerve with radius $R=900\mu$, the ICP must be at least 7.4 mmHg above normal for the axonal displacement characteristic of papilledema to occur. Thus, an infinitesimal rise in CSF pressure will not cause papilledema. Papilledema will develop only if CSF pressure rises sufficiently for axoplasmic displacement to take place.

### Resolution of Critique 3.3

We have shown that compression of the retrolaminar optic nerve produces two important stresses—an axial gradient of tissue pressure and a shear stress acting on planes perpendicular to the radial coordinate. The gradient of tissue pressure acts to displace axoplasm from the extraocular to the intraocular portion of the axon, and the shear stress acts to disrupt the cytoskeletal network and interfere with axonal transport. These two forms of stress share some common features. Both are proportional to the degree of external compression. Both increase in magnitude with radial distance from the center of the nerve cross-section. In the axial direction both are maximal at the point (or zone) of transition from compressed to uncompressed nerve. However, there are also differences, so that one cannot conclude that they are merely two sides of the same coin. The peripheral predominance in the cross section of the nerve is much more pronounced for the maximum gradient of tissue pressure than for the maximum shear stress (compare Fig. 3 with Fig. 4). Also, the axial tissue pressure gradient induces axoplasmic displacement more easily in large diameter axons than in smaller diameter axons, because it is the product of the gradient and the axon radius that must exceed twice the yield stress of the axoplasmic gel. By contrast, the effectiveness of the shear stress in disrupting the axonal cytoskeleton has no clear dependence on axon diameter.

These differences between the two stresses may account for the wide variation in axon morphology seen in papilledema—in particular, the lack of a tight correlation between intraaxonal organelle accumulation and degree of swelling of the axon. The organelle accumulation is consistent with blocked axonal transport. The swelling, however, is the result of retrograde axoplasmic displacement. The currently prevailing theory predicts that the degree of axonal swelling should be proportional to the degree to which axonal transport is blocked, since the latter causes the former. In contrast, the present hypothesis, although it allows for a positive correlation between blocked transport and extent of swelling (since both forms of stress are proportional to the degree of external compression and both share peripheral radial predominance), also allows for some degree of independence between the effects of the two forms of stress. Thus, one might find some very peripheral, relatively large diameter axons that show extreme swelling without evidence of blocked axonal transport, since the shear stress ${\tau }_{rz}$ falls to zero at the extreme periphery. Or there may be axons sufficiently far from the center of the cross-section to have considerable shear stress but not far enough or large enough to generate retrograde axial displacement. They would have accumulations of mitochondria and other organelles but show little or no swelling.

### Limitations of this Study

Our study has several limitations. First, as noted previously, our model of the optic nerve as a long cylinder, stretching in both directions from a transition point between compressed and uncompressed nerve, is anatomically inaccurate. While it accurately portrays what we have called region 1 and region 2 of our model, it ignores the fact that the prelaminar nerve extends only a short distance from the transition point before entering the nerve fiber layer on the inside wall of the spherical shell that constitutes the retinal surface of the eye. Thus region 3 is the inner surface of a spherical shell and not a solid cylinder. However, it is notable that the axons of region 3 are not compressed by external forces (elevated ICP) and remain unstressed by the external forces in regions 1 and 2. We thus believe that this inaccuracy does not significantly affect our conclusions, because, being both uncompressed and unstressed, the course of the axons in the retinal nerve fiber layer is not relevant with regard to the distribution of stresses in regions 1 and 2.

A second limitation of our model is that we used equations of continuum mechanics that assume linear and isotropic behavior of the object of study. As to linearity, the displacements of neural tissue in papilledema are of such a size as to cast doubt on the linearity of the stress–strain relationships. Also, as mentioned earlier, the optic nerve itself, as well as the axoplasm within its axons, consist primarily of long tubular and filamentous structures and is thus anisotropic. Given these deviations from linearity and isotropy, one cannot expect results that have a high degree of accuracy. Yet, that was not our intention. Our goal was to determine the qualitative behavior of the compressed nerve with sufficient confidence to formulate an alternative to the prevailing hypothesis.

The equations of continuum mechanics that we have used also assume that the object of study is homogeneous. The object of our study, the intraocular and immediately extraocular optic nerve, is not homogeneous. In particular, the presence of the LC in the posterior half of the intraocular portion of the nerve is a notable inhomogeneity. Absent from the prelaminar optic nerve tissue, the successive meshes of connective tissue that constitute this structure begin at the level of the retina and increase in prominence as the nerve traverses its intraocular course towards the transition point. There have been no prior experimental studies or biomechanical models of papilledema that might shed light on the effect that the LC would have on our results. However, the LC has been the object of interest in numerous biomechanical studies in the field of glaucoma research (Belleza et al. 2000; Dongqi and Zeqin 1999; Edwards and Good 2001; Sigal and Ethier 2009; Sigal et al. 2004). The interest derives from an early theory of the pathogenesis of glaucoma, which asserts that increased IOP damages neural tissue by means of displacing the LC posteriorly, shearing the axon bundles running through its pores (Birnbacher and Czermak 1885, 1886). Advocates of this theory point to the posterior border of the LC as the site of blockage of axonal transport and the locus of early axonal damage. However, this theory has been strongly contested by numerous students of acute glaucoma (Elschnig 1928; Schnabel 1905; von Hippel 1910), who have noted severe displacement or loss of neural tissue with no changes in the position of the LC. In their view the primary effect of acute elevation of IOP is neural damage. In fact, some believe that displacement of the LC is largely a secondary effect of increased IOP, facilitated by loss of the neural and glial tissue that otherwise support it. Our results provide indirect evidence for the view that the damage to neural tissue from elevated IOP is direct and does not require the presence of the LC. We have found shear stress that can disrupt neural tissue and a strong gradient of hydrostatic tissue pressure that can displace axoplasm, which are sharply localized to a narrow zone of abrupt change in the degree of external compression. While this location coincides with that of the posterior LC, the stresses occurred in a model which assumed homogeneity; i.e., no lamina cribrosa.

Finally, our rheology relied on squid axoplasm, and it is not clear that mammalian axoplasm behaves similarly. For our conclusions, however, the precise yield stress of the axoplasmic gel and the viscosity of the liquified axoplasm are not crucial, and it is likely that mammalian axoplasm, although it may differ from that of the squid in these characteristics, still behaves like a Bingham body.

### Conclusions

Our analysis supports the widely held belief that papilledema is the mechanical consequence of a pathological difference in pressure between the CSF surrounding the retrolaminar optic nerve and the intraocular pressure. However, it does not support the linear chain of causation of the prevailing theory, whereby the external compression causes blockage of axonal transport which in turn causes a damming back of the axoplasm and thereby swelling of the prelaminar axons. Instead, we find that the external compression produces two types of stress in the optic nerve: 1. The tissue pressure, which is the negative of the average of the three normal stresses, has a steep gradient at the transition point which drives the displacement of axoplasm. The direction of the displacement depends on the direction of the gradient. If ICP is higher than IOP, the displacement will be from the extraocular to the intraocular segment, as in papilledema. If IOP is higher than ICP, the displacement will be from intraocular to extraocular, as in acute glaucoma. We have shown that the volumetric flow of the displaced axoplasm is consistent with the degree of axonal swelling in papilledema and its rate of development. 2. The axial shear stress, which is also highly localized to the region of the transition between compressed and uncompressed nerve, is optimally situated to disrupt the axonal cytoskeleton and interfere with axonal transport. We have argued that although both forms of stress are caused by the external compression of the retrolaminar nerve, their effects on axoplasmic displacement and blocked axonal transport need not be tightly yoked, since the magnitudes of the two forms of stress differ in their spatial distribution across the cross-section of the nerve, and in the dependence of their effects on the diameter of the axon. These differences may explain the wide variety of morphological changes observed in the prelaminar axons of papilledema.

## Data Availability

Data sharing is not applicable, as no new data were created or analyzed in this study.

## Appendix

To determine the stresses in a solid circular cylinder subject to forces on its surface that depend only on position along its length and not on position along its circumference requires us to solve the equations of equilibrium for an axisymmetric structure. When we apply the laws of linear elasticity, these are

8 $$\frac{\partial {\sigma }_{r}}{\partial r}+\frac{\partial {\tau }_{rz}}{\partial z}+\frac{{\sigma }_{r}-{\sigma }_{\theta }}{r}=0.$$

9 $$\frac{\partial {\tau }_{rz}}{\partial r}+\frac{\partial {\sigma }_{z}}{\partial z}+\frac{{\tau }_{rz}}{r}=0.$$

In these equations ${\sigma }_{r}\left(r,z\right)$ is the radial normal stress, ${\sigma }_{\theta }\left(r,z\right)$ is the hoop stress, ${\sigma }_{z}\left(r,z\right)$ is the axial normal stress; and ${\tau }_{rz}\left(r,z\right)$ is the only non-zero shear stress.

To solve these equations, we find a suitable stress function $\Phi \left(r,z\right)$. Any function $\Phi$ of $r$ and $z$ that satisfies the biharmonic equation

10 $$\nabla^{2} \nabla^{2} \Phi \left( {r,z} \right) = 0$$

will satisfy the equilibrium equations $(8)$ and $(9)$ if we set:

11 $$\sigma_{r} = \frac{\partial }{\partial z}\left( {\nu \nabla^{2} \Phi - \frac{{\partial^{2} \Phi }}{{\partial r^{2} }}} \right)$$

12 $$\sigma_{\theta } = \frac{\partial }{\partial z}\left( {\nu \nabla^{2} \Phi - \frac{1}{r}\frac{\partial \Phi }{{\partial r}}} \right)$$

13 $$\sigma_{z} = \frac{\partial }{\partial z}\left( {\left( {2 - \nu } \right)\nabla^{2} \Phi - \frac{{\partial^{2} \Phi }}{{\partial z^{2} }}} \right)$$

14 $$\tau_{rz} = \frac{\partial }{\partial r}\left( {\left( {1 - \nu } \right)\nabla^{2} \Phi - \frac{{\partial^{2} \Phi }}{{\partial z^{2} }}} \right)$$

where the parameter $\nu$ represents the Poisson ratio.

Of the many stress functions solving the biharmonic equation, we must find the one that will yield stresses that satisfy the boundary conditions of our problem. These are:

15 $$\tau_{rz} = 0\quad \text{at}\;r = R,$$

where $R$ is the radius of the cylinder, and

16 $$\sigma_{r} = - P\;\text{for}\;z < 0\quad {\text{and}}\quad\sigma_{r} = 0\;\text{for}\;z > 0,$$

where $z=0$ marks the transition point between the cylinder compressed by pressure $P$ ($z<0$) and the cylinder subject to no surface forces ($z>0$).

Following Timoshenko and Goodier (Timoshenko & Goodier 1970), if we assume a stress function of the form

$$\Phi =\text{g}\left(r\right)\,\,\text{cos}\,\,kz$$

and substitute into the harmonic equation ${\nabla }^{2}\Phi =0$, we find that $\text{g}\left(r\right)$ must satisfy the modified Bessel equation of order zero:

$$\frac{{d}^{2}g}{d{r}^{2}}+\frac{1}{r}\frac{dg}{dr}-{k}^{2}g=0,$$

which has a solution $\text{g}\left(\text{r}\right)= {I}_{o}(kr)$, where ${I}_{o}(kr)$ is the modified Bessel function of the first kind, order zero, and argument $kr$. As ${I}_{o}(kr)$ solves the harmonic equation, it also solves equation (10), the biharmonic equation. Another solution to the biharmonic equation can be obtained by substituting the function $kr{I}_{1}\left(kr\right)$ into the harmonic equation, where ${I}_{1}\left(kr\right)$ is the modified Bessel function of the first kind of order one. The result is a multiple of ${I}_{o}(kr)$, which, upon a second application of the harmonic operator equals zero, as noted above. Taking a linear combination of these two solutions, we get

$$\Phi = \left( {b_{0} I_{o} \left( {kr} \right) - b_{1} krI_{1} \left( {kr} \right)} \right) \cos kz,$$

where $b_{0}$, $b_{1}$ and $k$ are arbitrary constants. Letting $b_{0} = \varrho b_{1}$, we get

$$\Phi = \left( {\varrho I_{0} \left( {kr} \right) - krI_{1} \left( {kr} \right)} \right)b_{1} \cos kz.$$

Since the above is valid for arbitrary $b_{1}$ and $k$, we can superimpose multiple such expressions by letting $b_{1} = f\left( k \right)$ and integrating over all $k$ to get the more general stress function.

17 $$\Phi = \mathop \smallint \limits_{0}^{\infty } \left( {\varrho I_{0} \left( {kr} \right) - krI_{1} \left( {kr} \right)} \right)f\left( k \right) \cos kz\,\, dk.$$

Using Eq. (14) to calculate the shear stress ${\tau }_{rz}$ with $\Phi$ given by Eq. (17), we find that,

$$\tau_{rz} = - \mathop \smallint \limits_{0}^{\infty } \left[ {krI_{0} \left( {kr} \right) + \left( {2 - 2\nu - \varrho } \right)I_{1} \left( {kr} \right)} \right]k^{3} f\left( k \right)\cos kz \,\,dk.$$

From boundary condition equation (15) we conclude that the expression inside the brackets of the integrand must be zero when $r=R$. This allows us to solve for $\varrho$.

18 $$\varrho \left(k\right)=2\left(1-\nu \right)+kR\frac{{I}_{o}\left(kR\right)}{{I}_{1}(kR)}$$

Having determined $\varrho$, we still must determine $f\left(k\right)$ in equation (17) to fully specify $\Phi$ and allow us to calculate the stresses from Eqs. (11–14). To do so, it will be convenient to modify the boundary condition equation (16) as follows;

$${\sigma }_{r}=-P/2\,\,\text{for}\,\,{z<0}\quad\text{and}\quad{\sigma }_{r}=P/2\,\,\text{for}\,\,z>0.$$

We will be able to recover the actual boundary condition Eq. (16) by superimposing a uniform compression ${\sigma }_{r}=-P/2$ over the entire cylinder.

Since

$${\int }_{0}^{\infty }\frac{\text{sin}kz}{k}dk=\left\{\begin{array}{c}\pi /2\,\it, \text{if}\,\, z>0\\ 0\,\it, \text{if}\,\, z=0\\ -\pi /2\,\it, \text{if}\,\, z<0\end{array}\right.$$

we have

19 $$\frac{P}{\pi }{\int }_{0}^{\infty }\frac{\text{sin}kz}{k}dk=\left\{\begin{array}{c}P/2\,\it, \text{if}\,\, z>0\\ 0\,\it, \text{if}\,\, z=0\\ -P/2\,\it, \text{if}\,\, z<0\end{array}\right.$$

which corresponds to our modified boundary condition. Substituting equation (17) into equation (11), we find that,

$$\sigma_{r} = - \mathop \smallint \limits_{0}^{\infty } \left[ {\left( {1 - 2\nu - \varrho } \right)I_{0} \left( {kr} \right) + \left( {kr + \varrho /kr} \right)I_{1} \left( {kr} \right)} \right]k^{3} f\left( k \right)\sin kz\, dk.$$

This will satisfy the modified boundary condition if:

$$- \left[ {\left( {1 - 2\nu - \varrho} \right)I_{0} \left( {kr} \right) + \left( {kr + \varrho /kr} \right)I_{1} \left( {kr} \right)} \right]k^{4} f\left( k \right) = P/\pi$$ when $$r = R$$.

Solving for $f\left(k\right)$, we get

20 $$f\left(k\right)=\frac{-P}{\pi \left[\left(1-2\nu -\varrho \right){I}_{0}\left(kR\right)+\left(kR+\varrho /kR\right){I}_{1}\left(kR\right)\right]{k}^{4}}$$

With $\varrho$ and $f\left( k \right)$ now determined, $\Phi$ is completely specified, and the stresses can be calculated from equations (11–14). We add to these the stresses induced by a uniform compression, which adds the stress $- P/2$ to $\sigma_{r}$ and to $\sigma_{\theta }$ but leaves $\sigma_{z}$ and $\tau_{rz}$ unaltered. The final equations for the stresses are:

21 $${\sigma }_{r}=-\frac{P}{2}-{\int }_{0}^{\infty }\left[\left(1-2\nu -\varrho \right){I}_{0}\left(kr\right)+\left(kr+\varrho /kr\right){I}_{1}\left(kr\right)\right]{k}^{3}f\left(k\right)\,\text{sin}kz\, dk$$

22 $${\sigma }_{\theta }=-\frac{P}{2}-{\int }_{0}^{\infty }\left[\left(1-2\nu \right){I}_{0}\left(kr\right)-\left(\varrho /kr\right){I}_{1}\left(kr\right)\right]{k}^{3}f\left(k\right)\, \text{sin}kz\,dk$$

23 $${\sigma }_{z}={\int }_{0}^{\infty }\left[\left(4-2\nu -\varrho \right){I}_{0}\left(kr\right)+kr{I}_{1}\left(kr\right)\right]{k}^{3}f\left(k\right)\, \text{sin}kz\, dk$$

24 $${\tau }_{rz}=-{\int }_{0}^{\infty }\left[kr{I}_{0}\left(kr\right)+\left(2-2\nu -\varrho \right){I}_{1}\left(kr\right)\right]{k}^{3}f\left(k\right)\, \text{cos}kz\, dk$$

The solutions in equations (21–24) agree with those of Barton (1941), who used a different method of solution and presented results in the form of infinite (Fourier) series. Unfortunately, these results are not conducive to the calculations needed below, which, however, can be readily performed with the Fourier integral representations of Eqs. (21–24) and the use of numerical integration.

The hydrostatic tissue pressure by definition is ${P}_{h}= -\frac{1}{3}\left({\sigma }_{r}+{\sigma }_{\theta }+{\sigma }_{z}\right).$ Substituting from Eqs. (21–23) gives

25 $${P}_{h}=\frac{P}{3}-\frac{2}{3}{\int }_{0}^{\infty }\left[\left(1+\upsilon \right){I}_{0}\left(kr\right)\right]{k}^{3}f\left(k\right)\, \text{sin}kz\,dk$$

The tissue axial hydrostatic pressure gradient is the derivative of the tissue pressure with respect to z

26 $$\frac{\partial {P}_{h}}{\partial z}=-\frac{2}{3}{\int }_{0}^{\infty }{\left[\left(1+\upsilon \right){I}_{0}\left(kr\right)\right]k}^{4}f\left(k\right)\, \text{cos}kz\, dk$$

For simplification and convenient visualization, we can express the above equations in a dimensionless format. We nondimensionalize the independent variables by letting $\overline{r} = r/R$ and $\overline{z} = z/R.$ We set $w = kR,$ a dimensionless variable of integration, and we nondimensionalize the stresses by dividing by$P$. Then

27 $$\frac{{\sigma }_{r}}{P}=-\frac{1}{2}-{\int }_{0}^{\infty }\left[\left(1-2\nu -\overline{\varrho }\right){I}_{0}\left(w\overline{r }\right)+\left(w\overline{r }+\overline{\varrho }/w\overline{r }\right){I}_{1}\left(w\overline{r }\right)\right]\overline{f }\left(w\right)\, \text{sin}w\overline{z }\, dw$$

28 $$\frac{{\sigma }_{\theta }}{P}=-\frac{1}{2}-{\int }_{0}^{\infty }\left[\left(1-2\nu \right){I}_{0}\left(w\overline{r }\right)-\left(\overline{\varrho }/w\overline{r }\right){I}_{1}\left(w\overline{r }\right)\right]\overline{f }\left(w\right)\, \text{sin}w\overline{z }\, dw$$

29 $$\frac{{\sigma }_{z}}{P}={\int }_{0}^{\infty }\left[\left(4-2\nu -\overline{\varrho }\right){I}_{0}\left(w\overline{r }\right)+w\overline{r}{I }_{1}\left(w\overline{r }\right)\right]\overline{f }\left(w\right)\, \text{sin}w\overline{z }\, dw$$

30 $$\frac{{\tau }_{rz}}{P}=-{\int }_{0}^{\infty }\left[w\overline{r}{I }_{0}\left(w\overline{r }\right)+\left(2-2\nu -\overline{\varrho }\right){I}_{1}\left(w\overline{r }\right)\right]\overline{f }\left(w\right)\, \text{ cos}w\overline{z }\, dw,$$

where

31 $$\overline{\varrho }\left( w \right) = 2 - 2\nu + w\frac{{I_{0} \left( w \right)}}{{I_{1} \left( w \right)}}$$

and

32 $$\overline{f}\left( w \right) = - \frac{1}{{\pi w\left[ {\left( {1 - 2\nu - \varrho } \right)I_{0} \left( w \right) + \left( {w + \varrho /w} \right)I_{1} \left( w \right)} \right]}}$$

The normalized hydrostatic tissue pressure becomes

33 $$\frac{{P}_{h}}{P}=\frac{1}{3}-\frac{2}{3}{\int }_{0}^{\infty }\left[\left(1+\nu \right){I}_{0}\left(w\overline{r }\right)\right]\overline{f }\left(w\right)\, \text{sin}w\overline{z }\, dw$$

and the normalized axial hydrostatic pressure gradient is

34 $$\frac{\partial }{\partial \overline{z}}\frac{{P }_{h}}{P} \, = \, -\frac{2}{3}{\int }_{0}^{\infty }\left[\left(1+\nu \right){I}_{0}\left(w\overline{r }\right)\right]w\overline{f }\left(w\right)\, \text{cos}w\overline{z }\, dw.$$

Setting the value of the Poisson ratio $\nu$ equal to 0.3, we integrated equations $(30)$ and $(33)$ numerically for three values of $\overline{r }$ to provide the curves shown in Fig. 2, expressing $\frac{{\tau }_{rz}}{P}$ and $\frac{{P}_{h}}{P}$ as functions of the normalized axial coordinate $\overline{z }$. Numerical integration of equations $(30)$ and $(34)$, after setting $\overline{z }=0$, provide the maximum values of the normalized shear stress $\frac{{\tau }_{rz}}{P}$ and the maximum values of the magnitude of the normalized tissue hydrostatic pressure gradient $\frac{\partial }{\partial \overline{z}}\frac{{P }_{h}}{P}$, as functions of the normalized radial coordinate $\overline{r }$. The graphs of these functions are depicted in Figs. 3 and 4 respectively.

The above represent the solutions for the internal stresses of the cylinder induced by an abrupt change of external compressive pressure from $P$ to $0$ at the point $\overline{z} = 0$. With these solutions available, it is straightforward to solve for the internal stresses induced by a ramp of external pressure which descends linearly from $P$ to 0 over a normalized span $\overline{\Delta }$ centered at $\overline{z} = 0$. Because the governing differential equations $\left( {10, 11 - 14 } \right)$ are linear, the solutions for the sum of two external compressive forces is the sum of the solutions for each of the individual compressions. If we denote an abrupt change in compression from $P$ to $0$ at the point $\overline{z} = 0$ by $G\left( {\overline{z}} \right)$ and the downward ramp compression from $P$ to $0$ by$H\left( {\overline{z}} \right)$, then

35 $$H\left( {\overline{z}} \right) = P + \left( {1/\overline{\Delta }} \right)\left( {\mathop \smallint \limits_{ - \infty }^{{\overline{z}}} G\left( {\overline{z} + \frac{{\overline{\Delta }}}{2}} \right) - G\left( {\overline{z} - \frac{{\overline{\Delta }}}{2}} \right)d\overline{z}} \right)$$

Since the stresses induced by $G\left( {\overline{z}} \right)$ are known, we need only specify $\overline{\Delta }$ in order to calculate the stresses induced by $H\left( {\overline{z}} \right)$. We have illustrated these modified solutions for $\overline{\Delta }{ } = .04$ by the curves with dashed lines in Figs. 3 and 4. For a cylinder with a radius of 900 $\mu$ such as a human optic nerve, $\overline{\Delta } = .04$ corresponds to a transition zone extending 18 $\mu$ to either side of the point $\overline{z} = 0$.

## Footnotes

1 In the periphery the tissue hydrostatic pressure on the uncompressed side of the nerve becomes transiently negative after which it rises gradually towards zero (Fig. 2c). A negative hydrostatic pressure means that its effect on a structural element such as a small cube of tissue is to dilate it uniformly rather than to compress it.

2 While this equation applies to an ideal fluid with zero viscosity, a similar tradeoff of forms of energy exists for fluids with viscosity, except that one must allow for viscous pressure loss with flow and thus expect lowered velocities.
